# Target-user preferences, motivations, and acceptance for a dialectical behaviour therapy smartphone application for eating disorders

**DOI:** 10.1007/s40519-024-01646-8

**Published:** 2024-02-27

**Authors:** Cleo Anderson, Matthew Fuller-Tyszkiewicz, Mariel Messer, Jake Linardon

**Affiliations:** 1 School of Psychology, Deakin University, 1 Gheringhap Street, Geelong, VIC 3220 Australia; 2 Center for Social and Emotional Development, Deakin University, Burwood, VIC 3125 Australia

**Keywords:** Eating disorders, Digital health, Dialectical behavioural therapy, Smartphone apps

## Abstract

**Purpose:**

Evidence shows that dialectical behavioural therapy (DBT) is efficacious for eating disorders (ED), yet few people have access to specialized treatments like DBT. Translating key DBT skills for delivery via a smartphone application may broaden the dissemination of evidence-based interventions. However, prior to developing a DBT-based app, it is crucial to gather information on target-user needs and preferences. Assessing overall acceptance and identifying predictors of acceptance, informed by the UTAUT framework, is also important. This process ensures not only a demand for such an app, but also that users receive content and features tailored to their needs.

**Method:**

This study aimed to understand target-user preferences of DBT-based apps for EDs by assessing willingness to engage, overall acceptance levels, and preferred functionality/content delivery modes (*n* = 326 symptomatic participants).

**Results:**

Eighty-eight percent indicated they would be willing to use a DBT-based ED app if it were available. Acceptance levels of a DBT app were high (64%), which was uniquely predicted by performance expectancy (perceptions of how beneficial an intervention is) and facilitating conditions (expectations of technological infrastructure and support in interventions) in path analysis. Content perceived as important to contain were emotion regulation techniques, tailored intervention strategies, and psychoeducation.

**Conclusion:**

Findings generate important information about target-user preferences of a DBT-based app for EDs, highlighting necessary design principles for apps of this kind.

*Level of evidence* Level V, cross-sectional descriptive study.

**Supplementary Information:**

The online version contains supplementary material available at 10.1007/s40519-024-01646-8.

## Introduction

Dialectical behavioural therapy (DBT) for eating disorders (EDs) is an evidence-based treatment based on the affect regulation model [1]. This model postulates that ED behaviours serve the function of regulating negative emotional experiences [1]. Thus, DBT aims to foster healthy coping strategies through the acquisition of emotion regulation, mindfulness, distress tolerance, and interpersonal skills, though the latter is commonly omitted from DBT protocols for EDs, given that establishing interpersonal skills has its own treatment approach for eating disorders: interpersonal psychotherapy. Randomized and non-randomized controlled trials (RCT) have documented the efficacy and effectiveness of DBT for EDs, including for binge eating disorder (BED) [2–4] and bulimia nervosa (BN) [2, 5]. Moreover, research shows that DBT may produce comparable effect sizes to traditional cognitive behavioural therapy (CBT) [3].

Despite the efficacy of DBT, specialized treatments like DBT are rarely accessed, as fewer than 25% of people with EDs seek or receive evidence-based care [6]. Numerous help-seeking barriers have been identified and can be distinguished into client-side and service-side barriers. Well-cited client-side barriers include the high cost of treatment, geographical constraints, privacy concerns, and perceived stigma associated with seeking help [7]. Service-side barriers include limited therapist availability, lengthy waitlists, and a lack of adherence to evidence-based treatment protocols by some clinicians [7]. Such help-seeking barriers call for innovations in treatment delivery, with smartphone app-based interventions (apps) serving as a viable solution to the widespread service gap.

Growing research is beginning to test the clinical utility of apps and how they may fit within the broader range of ED treatments available through the public and private healthcare sectors. Apps show promise as a viable first step in care or as an adjunct to traditional face-to-face therapy for several reasons. First, in Australia, approximately 89% of people own a smartphone, with most taking their phones with them when they leave the house [8]. As such, apps can promote the integration of evidence-based intervention strategies into users’ everyday lives, allowing for greater skill utilization and acquisition. Second, apps enable efficient and user-friendly solutions for real-time symptom monitoring, rather than traditional pencil and paper methods that are often completed retrospectively. Data generated from real-time symptom monitoring can provide tailored and context-appropriate intervention content depending on the users’ progress, needs, and preferences [9]. Third, apps overcome several barriers to help-seeking in that they are inexpensive, can be engaged from any location at a time that is convenient to the individual and do not require ongoing support from a professional. Importantly, apps appear to be in demand by target-users, with one in three endorsing a preference for digitally delivered interventions over face-to-face treatment [10].

A growing number of apps for ED symptoms have been developed and evaluated in RCTs. Existing apps are largely based on traditional CBT principles, which principally aims to normalize eating patterns and restructure beliefs about the importance of weight and shape. RCTs show that these apps produce moderate to large improvements in numerous symptom measures among people with or at risk for EDs [11, 12]. For example, authors found that in symptomatic individuals (e.g., those who engaged in binge eating), a CBT app-delivered intervention resulted in significantly greater reductions in ED symptomology compared to those in the waitlist control group (*d* = − 0.8) [11]. Apps may also bolster the effectiveness of face-to-face ED treatment, with research demonstrating that, in men and women diagnosed with BED or BN, apps utilized as an adjunct to face-to-face treatment are superior to when face-to-face treatment is delivered alone [13].

Despite significant progress in developing and testing evidence-based apps for EDs, one limitation concerns the narrow variety in the type of content offered, as all are based on traditional CBT principles. However, evidence indicates that around 50% of people do not respond to CBT [14]. Possible reasons for this relate to its change-oriented approach that may be too confrontational for some or its limited focus on the role of emotion in EDs—a major risk and maintaining factor for ED behaviours [15]. Therefore, broadening the type of content offered in apps by utilizing different theoretical models and therapeutic techniques (e.g., DBT, which, while it can be perceived as somewhat change-oriented, is much less so than CBT and has a greater focus on emotion) is a fruitful avenue for future research, as it could provide more diverse content and aid those who do not want to receive CBT or who do not respond to it. While one evidence-based app, Recovery Record [16], contains some DBT features, it is primarily a self-monitoring app linked with clinicians. It lacks structured psychoeducation aligned with the DBT change model and was developed almost a decade ago, making it less adaptable to current technological advancements. Attempting to reshape this app could detract from its original purpose of self-monitoring. Instead, the focus should be on developing a variety of options, allowing users to choose apps and associated features tailored to their preferences and needs. This focus is crucial, given research findings indicating that aligning self-identified preferences and needs correlates with enhanced therapeutic outcomes [17]. New technology also allows apps to be built in a manner that supports ongoing updates, ensuring alignment with emerging evidence-based guidelines. Furthermore, there is the possibility of open-sourcing these apps, fostering global collaboration and advancing research and clinical practices in digital health. These factors collectively provide a rationale for developing new apps in the context of eating disorders, which would offer diverse content and up-to-date technological features, addressing existing limitations and aiming to enhance therapeutic outcomes.

Prior to developing and evaluating any technological intervention, it is crucial that information pertaining to target-user needs and preferences is gathered. Such information may confirm that a demand for an app based on DBT exists and is thus worth investing resources in developing, testing and disseminating it. It would also inform critical design choices by ensuring that target-users receive content and functionality that they believe will be beneficial, thus potentially enhancing engagement over longer periods. It is also essential the researchers investigate target-users’ acceptance of prospective apps and understand what factors are associated with target-users’ acceptance levels. According to the UTAUT framework [18], acceptance is the most proximal indicator of actual use, and there are several theoretical drivers of acceptance, including performance expectancy, effort expectancy, social influence and facilitating conditions. Recent research suggests that these drivers predict acceptance via individuals’ attitudes toward technology [19]. Understanding acceptance and its predictors is important for understanding how it may be plausible to foster acceptance of an app intervention in the target population.

### Summary and aims

The current study seeks to examine the target-user preferences, motivations and acceptance of an app based on DBT skills training for EDs. Specifically, four key aims have been formed. The first is to assess the degree to which target-users are willing to use an app based on DBT for ED symptoms. The second aim is to examine the extent to which target-users prefer a DBT versus a CBT-based app. The third aim is to assess the overall acceptance of an app based on DBT and to identify predictors of acceptance. To achieve this, we will evaluate the UTAUT model in the context of a DBT-based app for EDs by testing whether performance expectancy, effort expectancy, social influence, and facilitating conditions predict acceptance and whether these associations are mediated by attitudes towards online interventions [18]. The fourth aim is to understand target-user preferences for specific functionality (e.g., digital diary, automated support) and content delivery modes (e.g., video, audio, text-based).

## Method

### Participants and procedure

Three hundred and eighty-one participants were recruited to participate in this cross-sectional online survey. Recruitment of participants occurred through advertisements distributed throughout the last author’s ED-related psychoeducational platform, which consists of an open-access website and associated social media account that displays passive educational material about eating disorders [20]. Those who gave consent to participate were taken to an online questionnaire delivered through Qualtrics, which was presented in a fixed order and took approximately 20 min to complete. To ensure the validity of responses and minimize the presence of “bots” on the data, we implemented several checks when cleaning and screening the data. These included identifying instances where respondents completed the survey multiple times, flagging individuals who completed the survey exceptionally quickly and detecting 'straight lining,' where respondents provided identical answers in matrix tables to expedite the survey. This study received ethics approval from Deakin University (ID: 2022-160).

Participants were eligible to complete the survey if they were 18 or over. Of the 381 respondents, we found that 86% reported the presence of at least one episode of objective binge eating over the past month. As a key focus of DBT is to directly target binge eating behaviour and given that we are interested in gathering information from potential target-users of a DBT-based app for EDs, we made the decision to restrict the analyses to the 326 participants who reported binge eating. However, we note that the inclusion vs exclusion of the 14% of participants who did not report binge eating in the past month had no discernible impact on our findings. There was a significantly higher proportion of females and individuals who were receiving treatment for disordered eating behaviours and/or body image problems among those who reported binge eating. No other demographic differences were present.

### Study variables

The full survey battery is presented in the Supplementary Materials.

#### Background variables

Participants were asked to indicate their age, sex, employment status, level of education, and current smartphone usage patterns.

#### Help-seeking

Participants were asked whether they were receiving professional help for ED symptoms, whether they had previously used a mental health app, and whether they were familiar with DBT. Each were assessed via single items, which were developed by the authors. Response options were ‘Yes’ or ‘No’.

#### Willingness to engage in a DBT app

Willingness was assessed by asking participants whether they would be willing to engage an app based on DBT principles to help address or prevent any disordered eating behaviours?”. Response options were ‘Yes”, ‘No’, or “I have not made up my mind yet’.^1^ This question was developed by the authors.

#### Preference for an app based on DBT vs CBT

Guided by prior work [21] but modified to make it relevant to the current study, participants were asked to indicate whether they would prefer to receive CBT or DBT through an app (or none) if they were to seek help for disordered eating behaviours now or in the future.

#### Acceptance of an app based on DBT

Acceptance was assessed using five items derived from an existing measure of acceptance designed for digital interventions for depression [22]. Each item was slightly adapted to make it applicable to an app based on DBT for ED symptoms (instead of “*I could imagine to try out an Internet-based intervention for mental health*” we modified the item to “*I could imagine trying out a DBT skills training app for these eating problems*”). Items were rated along a 5-point Likert scale with responses ranging from 1 (*totally disagree*) to 5 (*totally agree*). Total scores were derived by summing each item, where higher scores indicate greater acceptance. Internal consistency has been shown to be acceptable across all subscales in previous research [23]. Internal consistency in this study was acceptable (*α* = 0.74).

#### Drivers of acceptance

As informed by the UTAUT model [18] four drivers of acceptance were also assessed. These included performance expectancy (i.e. a person’s perception of how beneficial an intervention is; 4 items), effort expectancy (i.e. the expected ease of using the intervention; 3 items), social influence (i.e. the perceived opinion of others in a person's social network regarding the intervention; 4 items) and facilitating condition (i.e. expectations of the technological infrastructure and support when using the intervention; 3 items). Each item was again modified to make it applicable to a DBT-based app and was rated on a scale ranging from 1 (*does not apply at all*) to five (*applies completely*). Internal consistency has shown to be acceptable across all subscales in previous research [23]. Cronbach’s alpha was alpha (a) in the present study 0.81, 0.59, 0.85, and 0.70, respectively. We also assessed attitudes towards a DBT-based app as a mediating variable [19]. Attitudes were measured using the 16-item Attitudes Towards Psychological Online Interventions (APOI) [24]. Each item was rated on a 5-point rating scale, ranging from 1 (*totally agree*) to 5 (*totally disagree*), and the sum of each item represented the total score, with higher scores reflecting greater reflecting more positive attitudes. Internal consistency has been established previously [24]. Cronbach’s alpha in the present study was 0.80.

#### Feature and content delivery preferences

Participants were presented with 20 distinct strategies, functions or features common to mental health apps (e.g., digital diary cards, graphs and charts). They were then asked to indicate how important (response options include ‘unimportant’, ‘neither important nor unimportant’ and ‘important’) it would be for each to be included within an app based on DBT. Similarly, participants were asked to indicate the importance (response options were ‘unimportant’, ‘neither important nor unimportant’ and ‘important’) of different modes of content delivery, including written text, audio recordings, video tutorials, pictures or graphics, or a mixture of these.

#### Eating disorder symptoms

The Eating Disorder Examination Questionnaire (EDE-Q) [25] was used to measure eating disorder symptomology. The EDE-Q comprises 28 self-report items which assess core cognitive and behavioural symptoms of EDs experienced over the previous 28 days (e.g., “has thinking about your shape or weight made it very difficult for you to concentrate on things you are interested in”). ED symptoms were assessed through the global score (average of the four subscales) and specific behavioural items (e.g., the frequency of binge eating and purging over the past 28 days), with higher scores reflecting more severe ED symptoms. Internal consistency, test–retest reliability, convergent validity, and incremental validity of the EDE-Q have been established previously [26]. Internal consistency in this study was acceptable for the total score (*α* = 0.92).

#### Emotion dysregulation

Emotion dysregulation was assessed via the abbreviated Difficulties in Emotion Regulation Scale (DERS-16) [27]. The DERS-16 consists of 16 items rated along a 5-point response scale, ranging from 1 (*almost never*) to 5 (*almost always*). The DERS-16 assess five dimensions of emotion dysregulation, including lack of emotion clarity (two items), limited access to emotion regulation strategies (five items), non-acceptance of negative emotion (three items), inability to engage in goal-directed behaviour under emotional distress (three items), and difficulties controlling impulsive behaviours (three items). A total score was derived by summing each dimension, and higher scores reflect greater emotion dysregulation. Internal consistency, test–retest reliability, and convergent and discriminate validity have been established previously [27]. Internal consistency in this study was acceptable for the total score (*α* = 0.95).

### Statistical analyses

**Fig. 1:**
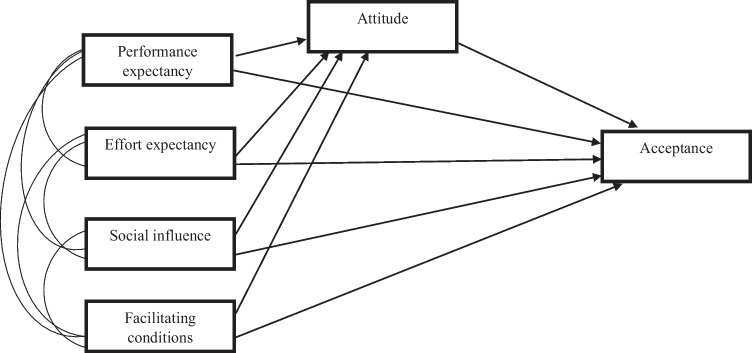
Proposed model of acceptance of DBT-based App for eating disorders

Analyses were conducted using IBM SPSS Statistics 29 and Mplus version 8.3. No missing data was present within the current study. For aims 1 and 4, basic descriptive statistics were computed. For aim 2, independent samples *t*-tests and Chi-square goodness-of-fit were used to compare group differences between those who selected a preference for a DBT versus a CBT-based app. For aim 3, a path analysis was computed to assess relationships between the four drivers of acceptance on acceptance levels and whether these relationships were mediated by attitudes (see Fig. 1). Maximum likelihood was employed as the estimator. Unstandardized regression weights and their associated significance levels were assessed for each pathway, in addition to the *R*^*2*^ values of each dependent variable. Bias-corrected bootstrap estimates (based on 1000 resamples) and 95% Confidence Intervals (CIs) were assessed to determine whether attitude mediates the relationship between the four drivers of acceptance and acceptance. If the CI did not contain zero, it indicated statistical significance.

## Results

### Sample characteristics

Two hundred and eighty-seven participants (88%) reported engaging in objective binge eating at least four times in the past month. Approximately one-quarter (26.4%) of participants reported receiving current ED treatment, and 38% reported having previous experience with a mental health app and had previous knowledge of DBT (Table 1).

**Table 1:** Participant characteristics

Variable	M (SD)
Age	36.75 (11.23)
ED symptomology	3.56 (1.19)
Number of binge-eating episodes	24.45 (27.66)
Emotion dysregulation	50.15 (15.20)

	*n* (%)
Gender (female)	295 (90.5%)
**Employment**	
Full-time	189 (58.0%)
Part-time	58 (17.8%)
Student	26 (8.0%)
Student and employed	31 (9.5%)
Not employed or student	22 (6.7%)
Education (tertiary)	230 (70.6%)
**Help-seeking**	
Current ED treatment (yes)	86 (26.4%)
Previous digital experience (yes)	124 (38.0%)
Knowledge of DBT (yes)	123 (37.7%)

ED symptomatology represents the EDE-Q global score. Emotion dysregulation represents the DERS total score

*ED* eating disorder, *DBT* dialectical behaviour therapy

### Willingness to engage in an app based on DBT

Most participants (88%) indicated a willingness to engage in an app based on DBT; 3.4% said they would not, and 8.6% stated that they were unsure.

### Preferences for a DBT versus a CBT-based app

Two hundred and thirty-four (71.8%) participants reported that they would prefer to receive an app based on DBT over CBT principles if they were to seek help for disordered eating symptoms. Only 2.8% reported that they were undecided.

Independent samples *t*-tests were computed to test for differences in those who reported a preference for an app based on DBT over CBT. The only significant difference (see Table 1 of the supplementary materials) to emerge was on the DERS total score, with those reporting a preference for DBT scoring significantly higher on emotion dysregulation than those reporting a preference for CBT (*t*(315) = 2.10, *p* = 0.04,* d* = 0.27).

### Acceptance levels

Participants’ mean score on acceptance for an app based on DBT was 16.04 (SD = 3.01). According to prior cut-offs [22], 64% of the sample was classified as having high acceptance (a score between 15–20), 32% had moderate acceptance (a score between 10–15), and 4% had low acceptance (scores < 9).

#### Path analysis

**Fig. 2:**
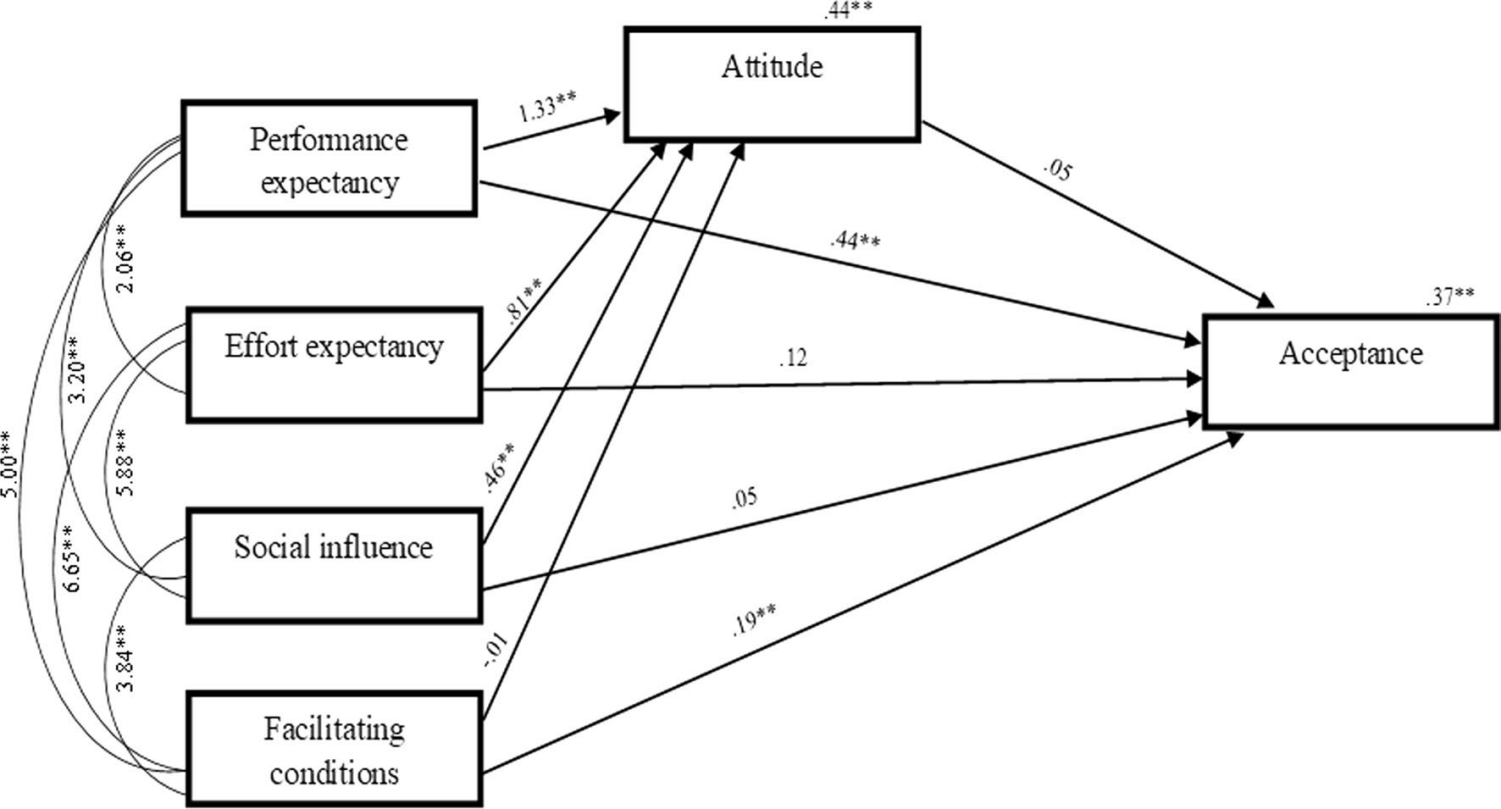
Path analysis model of the four key drivers of acceptance, attitude, and acceptance. Unstandardized coefficients are reported. **≤ 0.01. No significant indirect effects of attitudes were observed for performance expectancy and acceptance, *B* = 0.06, 95%CI [− 0.00, 0.13]; effort expectancy and acceptance, *B* = 0.04, 95%CI [− 0.00, 0.08]; facilitating conditions and acceptance *B* = 0.00, 95%CI [− 0.02, 0.02]; and social influence and acceptance* B* = 0.02, 95%CI [0.00, 0.05]

Path analysis tested whether performance expectancy, effort expectancy, social influence and facilitating conditions predicted acceptance levels via participant attitudes. Figure 2 depicts the unstandardized regression weights of the path analysis (Supplementary materials present correlations). Combined, the predictors explained 37% of the variance in acceptance. The only variables to uniquely predict acceptance were performance expectancy and facilitating conditions, with higher scores on these constructs predicting higher acceptance levels. No mediation effects emerged.

### Feature content delivery preference ratings

Of the 20 functionality items presented to participants (Table 2), those that received the highest importance endorsement ratings were ‘emotion regulation strategies ‘(96%) and ‘automated tailoring based on emotional state’ (92%). Those that received the lowest importance endorsement ratings were ‘social forums to connect with other users (33%)’ and ‘information sharing with health professionals’ (49%). The most frequently endorsed mode of delivery was a mixture of written text, video tutorials, audio recordings and pictures or graphics (85% endorsed as ‘important’).

**Table 2:** Functionality and mode of delivery preference ratings

	*n* (%) endorsing as ‘important’
**Specific functionality**	
General information on EDs	277 (85.0%)
Social forums to connect with other users	107 (32.5%)
Rewards for completing tasks	207 (63.5%)
Goal-setting	282 (86.5%)
Progress graphs/charts	269 (82.5%)
Diary card for thoughts, feelings, and behaviours	220 (67.5%)
Push notifications/reminders to use the app daily	178 (54.6%)
Sensor functionality to tailor strategies	168 (51.5%)
Social relationship improvement strategies	205 (62.9%)
Information sharing with health professional	161 (49.4%)
Automated progress feedback	254 (77.9%)
Automated service/chat-bot	165 (50.6%)
Further support options	169 (51.8%)
Emotion regulation strategies	312 (95.7%)
App usage history	249 (76.4%)
Feedback from professionals	254 (74.8%)
Automated tailoring	300 (92%)
Quizzes	192 (58.9%)
Guided mindfulness exercises	232 (71.2%)
Screening to assess ED levels	232 (71.2%)
**Mode of delivery**	
Video	163 (50%)
Images/graphics	266 (82%)
Written text	260 (80%)
Audio	102 (31%)
Mixture	266 (85%)

*ED* eating disorder

## Discussion

### Main findings

Several important findings emerged. First, most participants (88%) indicated that they would be willing to use a DBT-based app in this context if it were available, with more preferring a DBT app over a CBT one. As expected, those who selected a preference for DBT exhibited higher emotion dysregulation scores, suggesting that such participants may have good insight into what might maintain their binge eating. Second, two-thirds of the sample reported high acceptance of a DBT app, with performance expectancy and facilitating conditions being the only unique correlates. Third, participants expressed preferences for specific features and content delivery formats, informing future design choices.

The present findings suggest potential receptivity within the target population toward an app based on DBT skills training for EDs. This suggests that developing, evaluating, and disseminating an app based on DBT principles may be worthwhile and could help broaden access to evidence-based treatment content for those in need or who cannot seek help via more traditional means. Unexpectedly, more participants expressed preference for a DBT over a CBT-based app, further highlighting the possibility that more people may be willing to access digital solutions if different options were available. A possible explanation for this finding may be derived from the difference in the core foundations of each approach, with many participants potentially finding CBT too limited in content for specific problem areas, especially emotion dysregulation [28].

We also identified two key correlates of acceptance levels. The first was performance expectancy, suggesting that those who expect personal benefit and improvement from an app are more likely to report an accepting stance toward app solutions. That performance expectancy was shown to predict acceptance suggests that further efforts to educate the public about the effectiveness of apps and their utility—as has been shown through acceptance-facilitating interventions [22, 23]—may be a viable way to enhance acceptance of a DBT app for EDs. The second predictor of acceptance was facilitating conditions, indicating that having high expectations of the technological infrastructure and support while using an app was associated with higher acceptance. Perhaps ensuring that target-users have technical support at hand for an app intervention will encourage acceptance levels, thus potentially leading to greater app uptake. One potential approach to achieving this is through a novel method called the ‘digital navigator’, which is an auxiliary care team member whose role is to support digital therapeutic alliance [29]. The digital navigator can help overcome technological barriers to app use by assisting individuals with the initial set-up of apps and troubleshooting technical problems [29]. The digital navigator shows promise as a possible solution to address technological barriers and facilitate the use of mental health apps in everyday life. Evaluating this in the context of ED treatment is worthwhile.

We explored preferences for functionality to be included within apps based on DBT for EDs. The most preferred functionality was strategies to tolerate emotions better. This finding is consistent with participants' preferences for DBT, which emphasize emotion regulation. Further, participants reported moderate difficulty in accessing emotion regulation strategies, perhaps explaining why many are interested in learning these strategies. Automated tailoring, such as systems driven by machine learning algorithms allowing for personalized guidance or prompts, was another highly endorsed function. This finding aligns with past research highlighting the added benefit of tailoring content in online interventions for depression [30] and anxiety [31]. Further, recent RCTs [32, 33] have demonstrated that individuals using apps with tailored suggestions based on their check-in assessment data experienced more substantial improvements in anxiety, experimental avoidance, cognitive fusion, psychological distress, and positive mental health compared to those receiving randomly suggested skills. Perhaps users desire this feature due to its ability to mirror clients’ experience of face-to-face treatment with a therapist, insofar as it enables regular “check-ins” with symptoms and tailors intervention content according to progress, needs, and preferences. Collectively, these findings may serve as a blueprint for developing DBT-based apps for EDs, highlighting important features, functions, and content delivery methods to include.

### Limitations

The current study has important limitations. First, our online recruitment method, which primarily involved social media sites, may have introduced the potential for selection bias such that we may have sampled individuals who regularly engage with technology and may be more familiar with apps than the general population. Second, as we only sampled adults, findings cannot be generalized to younger individuals with ED symptoms. It is possible that preference ratings and attitudes differ as a function of distinct age groups. Further, most of the sample identified as female and therefore, the findings cannot be generalized to other gender orientations. Third, the current study did not measure the actual usage of a smartphone app of this nature. While acceptance is considered the best indicator of actual usage, the extent to which stated intentions align with actual behaviour remains unclear and should be investigated further. Fourth, given that only short descriptions were presented to participants, it may be the case that individuals did not understand the differences between DBT and CBT, potentially impacting the validity of these responses. Further, participants were asked to rate levels of acceptance and preferences based solely on these descriptions of an app that has not yet been created or visually presented to them, meaning that the study remains on a hypothetical level. Fifth, two subscales (effort expectancy and facilitating conditions) exhibited low internal consistency, which should be kept in mind when interpreting the present findings. As the original scales assessing these constructs were adapted from other fields to make it applicable to digital mental health, it would be worthwhile for future research to develop a psychometrically sound measure of acceptance and its hypothesized drivers of app-based mental health interventions. Sixth, despite drawing from validated scales assessing technological acceptance, attitudes, and preferences, we were required to re-word several items to make them specific to the context of the present aims of this research. This should be kept in mind when interpreting the present findings. Seventh, the current study did not conduct qualitative interviews in order to capture nuanced user-needs. Conducting in-depth interviews with target-user to gain further insight into the receptiveness of an app of this nature is an important next step, particularly now that there is evidence that a demand is present. Finally, considering that apps can serve both preventive and treatment purposes, the current sample included individuals exhibiting disordered eating symptoms rather than exclusively focusing on diagnosed cases. Therefore, it should be noted that the current study does not gain insight into the preferences of individuals with a diagnosable ED, nor can the findings be generalized to individuals without any disordered eating symptoms. Future research may be interested in gathering the perspectives of these specific populations.

## Conclusions and future directions

The current study gathered target-user information regarding the needs and preferences for an app based on DBT for EDs. We found that symptomatic individuals are receptive and accepting of an app based on DBT principles, with more expressing a preference for DBT over a CBT-based app. Further, our findings revealed that certain drivers of acceptance may be more influential than others. Given the high prevalence of co-morbidity in EDs with suicidality and self-harming behaviours [34] and preliminary evidence indicating that emotion dysregulation is a salient factor in examining suicidality and EDs [35], exploring the needs of individuals dealing with such co-morbid conditions could be an important next step for new or even existing apps based on this therapeutic approach (e.g., DBT coach). For example, investigating the potential inclusion of routinized risk assessments in apps to help manage these co-occurring behaviours alongside eating behaviours may be a valuable direction for future studies. Overall, our results provide much-needed information for experts interested in developing an app for EDs based on DBT.

### What is already known on this subject?

Previous research has explored target-user preferences for digital interventions in the context of EDs. However, little is known about the reception of DBT-based apps specifically for this population. We addressed this gap.

### What this study adds?

This is the first study to gather target-user information regarding the needs and preferences for an app based on DBT for EDs. We found that symptomatic individuals are receptive and accepting of an app based on DBT principles, with more expressing a preference for DBT over a CBT-based app. Further, our findings revealed that certain drivers of acceptance may be more influential than others. Overall, our results provide much-needed information for experts interested in developing an app for EDs based on DBT.

### Supplementary Information

Below is the link to the electronic supplementary material.

Supplementary file1 (DOCX 31 KB)

## Data Availability

Non-identifiable data can be made available on request.

## Footnotes

1 Participants were provided with a description of both the CBT and DBT approach at the start of the survey that was matched in length to ensure that participants were able to answer the items in the survey battery. This ensured that participants understood specific questions pertaining to either CBT or DBT. The description provided to participants was as follows: “Dialectical behavioural therapy (DBT) skills training acknowledges that our emotional reactions play a major role in contributing to disordered eating behaviours, like binge eating, dietary restriction and purging. DBT aims to target these disordered eating behaviours by teaching people how to effectively tolerate periods of emotional distress, improve the quality of interpersonal relationships, be more mindful of their surroundings, and use adaptive emotion regulation strategies. Cognitive-behavioural therapy (CBT) focuses on the close relationships between our thoughts, feelings, and behaviours. It teaches the person to recognize how their beliefs and feelings about body image and dieting contribute to disordered eating behaviours. CBT aims to change these disordered eating patterns directly via behavioural experiments and encourages the person to challenge and overcome unhelpful beliefs about eating, body image and dieting.”
