# Supplementary material for: Target-user preferences, motivations, and acceptance for a dialectical behaviour therapy smartphone application for eating disorders
Source: Eat Weight Disord. 2024 Feb 27;29(1):17. doi: 10.1007/s40519-024-01646-8 (PMC10899409; doi:10.1007/s40519-024-01646-8)
Supplement: Supplementary file 1 — Supplementary file1 (DOCX 31 KB) [file 40519_2024_1646_MOESM1_ESM.docx]

**Additional Materials**

**Table S1.**

***Univariate analyses***

| Variable | DBT | CBT | Test Statistic | | ES |  |
| --- | --- | --- | --- | --- | --- | --- |
|  |  |  | t | χ *^2^* |  | |
| Age, *M(SD)* | 36.090 (10.562) | 37.891 (12.17) | -1.282 |  | -.164 | |
| Gender, *n* (%) female | 213 (91%) | 74 (89%) |  | .617 | .617 | |
| Employment, *n* (%) |  |  |  | 8.53 | .164 | |
| Full time employment | 132 (56.4%) | 54 (65.1%) |  |  |  |  |
| Part-time employment | 44 (18.8%) | 12 (14.5%) |  |  |  |  |
| Student | 15 (6.4%) | 10 (12%) |  |  |  |  |
| Student and employed | 28 (12%) | 3 (3.6%) |  |  |  |  |
| Not employed or student | 15 (6.4%) | 4 (4.8%) |  |  |  |  |
| Education, *n* (%) tertiary | 166 (71%) | 60 (72.3%) |  | .05 | .01 | |
| Current ED treatment, *n* (%) yes | 68 (29%) | (19%) |  | 3.01 | .097 | |
| Previous digital experience, n (%) yes | 95 (41%) | 28 (34%) |  | 1.22 | .06 | |
| Knowledge of DBT, n (%) yes | 96 (41%) | 25 (30%) |  | 3.09 | .10 | |
| ED Symptomology, *M(SD)* | 3.58 (1.20) | 3.71 (1.14) | -.855 |  | -0.110 | |
| DERS Total Score, *M (SD)* | 51.47 (14.39) | 47.45 (16.52) | 2.10* |  | .269 | |
| *= statistically significant at the *p* < .05 level. | | | | | |  |

**Table S2.**

*Bivariate correlations between all key variables included within the model of acceptance.*

| **Variable** | **1** | **2** | **3** | **4** | **5** | **6** | |
| --- | --- | --- | --- | --- | --- | --- | --- |
| 1. PE |  |  |  |  |  |  | |
| 1. EE | .366** |  |  |  |  |  | |
| 1. SI | .436** | .310** |  |  |  |  | |
| 1. FC | .322** | .342** | .201** |  |  |  | |
| 1. Attitude | .606** | .430** | .432** | .252** |  |  | |
| 1. Acceptance | .566** | .342** | .325** | .335** | .450** |  | |
| Mean | 15.36 | 10.99 | 13.87 | 12.77 | 56.29 | 16.04 | |
| SD | 2.73 | 2.07 | 2.70 | 2.23 | 7.89 | 3.01 | |
| α | .81 | .59 | .85 | .69 | .80 | .74 | |
| Note. PE = Performance expectancy, EE = effort expectancy, SI = social influence, FC = facilitating condition. ** = Correlation is significant at the 0.01 level (two-tailed). | | | | | | |  |

**Survey Items**

1. Are you over the age of 18?
2. Yes
3. No

2. What is your age in years?

3. What is your gender?

a. Male

b. Female

c. Other

4. What is your highest level of education?

a. No school certificates

b. Secondary school

c. Post-secondary diploma/certificate/associates degree

d. Undergraduate degree

e. Master’s degree

f. Ph.D

g. Other degree (please specify)

5. In which country do you reside? (please specify)

6. What is your current employment status?

a. Full time

b. Part-time

c. Student

d. Not currently working

7. Are you currently receiving professional help for disordered eating behaviours and/or body image problems?

a. Yes

b. No

8. Have you had any prior experience with or used a digital psychological intervention (i.e., interventions delivered via a computer, mobile phones apps, or tablet)?

a. Yes

b. No

9. Do your currently own a smartphone?

a. yes

b. no

10. Do you currently have regular access to the internet?

a. yes

b. no

14. Have you ever heard or read about dialectical behaviour therapy or dialectical behaviour therapy skills training?

a. Yes

b. No

**Digital Psychological Intervention**

**Definitions**

Below we are going to present you with a description of two common psychological interventions for disordered eating that can be delivered via digital technology, like the internet or smartphone app. Please read these descriptions carefully.

1. Digital dialectical behaviour therapy skills training: DBT skills training acknowledges that our emotional responses play a major role in contributing to disordered eating behaviours, like binge eating and dietary restriction. Therefore, DBT aims to break the cycle of disordered eating by teaching people how to regulate their emotional reactions in a more adaptive way. In doing so, DBT teaches strategies that help us live in the moment, cope with stress in healthier ways, and to improve the quality of relationships with others.
2. Digital cognitive-behavioral therapy: CBT is an approach that focuses on the relationships between our thoughts, feelings, and behaviours. It is an approach that will help you to recognize how your beliefs about the importance of body image and dieting directly contribute to negative mood states and disordered eating behaviours. CBT teaches strategies that help the individual change their unhealthy eating behaviours directly and challenge and overcome unhelpful beliefs about eating, weight and shape.

**Intervention Preference**

1. If you were to seek help for any current or future disordered eating (which are defined as behaviours such as grazing, obsessive dieting or fasting, loss of control eating, compulsive exercise, laxative misuse, or self-induced vomiting), which of the three options would you most prefer to receive (choose one)?

a. Digital DBT skills training

b. Digital CBT-based intervention

c. I wouldn’t want to receive any of these.

**Acceptance of a DBT digital intervention**

DBT skills training acknowledges that our emotional responses play a major role in contributing to disordered eating behaviours, like binge eating and dietary restriction. Therefore, DBT aims to break the cycle of disordered eating by teaching people how to regulate their emotional reactions in a more adaptive way. In doing so, DBT teaches strategies that help us live in the moment, cope with stress in healthier ways, and to improve the quality of relationships with others.

Based on the description of the digital DBT program above, please indicate the degree to which you agree with the following statements using the following response scale;

a. totally disagree

b. slightly disagree

c. neither agree nor disagree

d. slightly agree

e. totally agree

"If I was exhibiting disordered eating symptoms, such as loss of control binge eating..."

1. "I could imagine trying out a digital DBT skills training intervention for these problems"

2. I would use a digital DBT skills training intervention, if offered"

3. "I would recommend a digital DBT skills training intervention to a friend"

4. "I would be willing to pay for a digital DBT skills training intervention for these problems

**Drivers of acceptance**

Please respond to the following responses using the following response scale:

a. totally disagree

b. slightly disagree

c. neither agree nor disagree

d. slightly agree

e. totally agree

**Performance expectancy**

1. Using a digital DBT skills training intervention would reduce my disordered eating.
2. Using a digital DBT skills training intervention for disordered eating would improve my personal wellbeing.
3. I would receive help for disordered eating from a digital DBT skills training intervention.
4. Using a digital DBT skills training intervention would help me to cope with disordered eating.

**Effort Expectancy**

1. Using a digital DBT skills training intervention for disordered eating would cost me a lot of time and energy.
2. Using a digital DBT skills training intervention would be an easy task for me.
3. A digital DBT skills training intervention for disordered eating would be clear and easily comprehensible to me.

**Social influence**

1. People close to me would recommend me to use a digital DBT skills training intervention.
2. My general practitioner would recommend me to use a digital DBT skills training intervention.
3. People close to me would use a digital DBT skills training intervention in case they would suffer from disordered eating.
4. Other people would think badly about me if I would use a digital DBT skills training intervention for disordered eating

**Facilitating conditions**

1. I have all the necessary technical preconditions for using a digital DBT skills training intervention for disordered eating.
2. In case of technical problems with a digital DBT skills training intervention, I would receive technical support.
3. I have the technical knowledge to utilize a digital DBT skills training intervention.

**Attitudes Towards an online DBT App**

The following statements deal with online DBT-based skills training interventions. Please state your personal appraisal based on the responses outlined below.

1. Totally agree
2. Rather agree
3. Not sure
4. Rather disagree
5. Totally disagree
6. By using an online DBT skills training intervention, I do not expect long-term effectiveness.
7. By using an online DBT skills training intervention, I do not receive professional help.
8. It is difficult to implement the suggestions of an online DBT skills training intervention effectively in everyday life.
9. Online DBT skills training interventions could increase isolation and loneliness.
10. An online DBT skills training intervention can help me recognize the issues that I have to challenge.
11. I have the feeling that an online DBT skills training intervention can help me.
12. An online DBT skills training intervention can inspire me to better approach my problems.
13. I believe that the concept of online DBT skills training intervention makes sense.
14. In crisis situations, a therapist can help me better than an online DBT skills training intervention.
15. I learn skills to better manage my everyday life from a therapist rather than from an online DBT skills training intervention.
16. I am more likely to stay motivated with a therapist than when using an online DBT skills training intervention.
17. I do not understand therapeutic concepts as well with an online DBT skills training intervention as I do with a therapist.
18. An online DBT skills training intervention is more confidential and discreet than visiting a therapist.
19. By using an online DBT skills training intervention, I can reveal my feelings more easily than with a therapist.
20. I would be more likely to tell my friends that I use an online DBT skills training intervention than that I visit a therapist.
21. By using an online DBT skills training intervention, I do not have to fear that someone will find out that I have eating behavior problems

**Intention to use digital DBT program**

Please respond to the following question by selecting ‘yes’, ‘no’, or ‘not sure’:

Would you be interested or willing to engage in a digital DBT skills training intervention target disordered eating behaviours?

a. Yes

b. No

c. Not sure

**Specific functionality preferences**

A list of features that are sometimes incorporated within digital interventions for disordered eating will now be presented.

Please indicate how important the following features are to you when considering a digital DBT-based skills training for binge-eating:

1. Not important at all
2. Slightly important
3. Moderately important
4. Very important
5. Extremely important
6. Screening scales to assess my level of disordered eating
7. Psychoeducational material teaching me about the nature of disordered eating
8. Digital diary to record my thoughts, feelings and behaviour
9. Automated feedback about my progress
10. Feedback from a trained professional on my progress
11. Social forums to connect with other users
12. Push notifications to remind me to keep engaging
13. Chat-bot to talk to if I feel stuck or lost
14. Automated prompts advising me on which strategies I should implement depending on where I am or what I am feeling in that moment
15. Rewards for completing required tasks (e.g., gamification principles)
16. Sensory functionality (e.g., heart rate monitor, smartwatch) to help deliver me tailored intervention content
17. Crisis support service
18. Interactive quizzes to test my knowledge
19. Goal setting exercises
20. Strategies to help me improve my relationships
21. Strategies to help me tolerate my emotions better
22. Strategies to help me become more mindful
23. Graphs & charts that will visualize my progress over time
24. Ability to share information with my healthcare professionals
25. History of my pass app use so I can recall where I am up to.

**Mode of Information Delivery Preference**

Digital intervention programs can present content in many formats, such as text, audio, images, and videos. If you were to use a DBT-based digital intervention for disordered eating, please indicate which of the following content formats you would prefer.

1. Written text only
2. Audio recordings only
3. Video presentations
4. A combination of these

**Emotion Regulation**

Please indicate how often the following statements apply to you by writing the appropriate number from the scale below (1-5).

1. I have difficulty making sense out of my feelings

2. I am confused about how I feel

3. When I am upset, I have difficulty getting work done.

4. When I am upset, I become out of control

5. When I am upset, I believe that I will remain that way for a long time

6. When I am upset, I believe that I'll end up feeling very depressed

7. When I am upset, I have difficulty focusing on other things

8. When I am upset, I feel out of control

9. When I am upset, I feel ashamed with myself for feeling that way

10. When I am upset, I feel like I am weak

11. When I am upset, I have difficulty controlling my behaviours

12. When I am upset, I believe there is nothing I can do to make myself feel better

13. When I am upset, I become irritated with myself for feeling that way.

14. When I am upset, I start to feel very bad about myself

15. When I am upset, I have difficulty thinking about anything else

16. When I am upset, my emotions feel overwhelming

**Eating Disorder Behaviour**

Instructions: The following questions are only concerned with the past four weeks (28 days). Please read each question carefully. Please answer all of the questions. Please only choose one answer for each question. Thank you.

Questions 1 to 12: Please circle the appropriate number on the right. Remember that the questions only refer to the past four weeks (28 days) only.

On how many of the past 28 days…

Response options:

- 1. No days
  2. 1-5 days
  3. 6-12 days
  4. 6-12
  5. 13-15
  6. 16-22
  7. 23-27
  8. Everyday

1. Have you been deliberately trying to limit the amount of food you eat to influence your shape or weight (whether or not you have succeeded)?
2. Have you gone for extended periods (8 waking hours or more) without eating anything at all in order to influence your shape or weight?
3. Have you tried to exclude from your diet any food that you like in order to influence your shape or weight (whether or not you have succeeded)?
4. Have you tried to follow definite rules regarding your eating (for example, a calorie limit) in order to influence shape or weight (whether or not you have succeeded)?
5. Have you had a definite desire to have an empty stomach with the aim of influencing your shape or weight?
6. Have you had a definite desire to have a flat stomach?
7. Has thinking about food, eating or calories made it very difficult to concentrate on things you are interested in (for example, working, following a conversation, or reading)?
8. Has thinking about shape or weight made it difficult to concentrate on things you are interested in (for example, working, following a conversation, or reading)
9. Have you had a definite fear of losing control overeating?
10. Have you had a definite fear of gaining weight?
11. Have you felt fat?
12. Have you had a strong desire to lose weight?

Questions 13-18: Please fill in the appropriate number in the number in boxes on the right. Remember that the question only refers to the past four weeks (28 days)

Over the past four weeks (28 days)

1. Over the past 28 days, how many times have you eaten what other people would regard as an unusually large amount of food (given the circumstances)?
2. ….On how many of these times did you have a sense of having lost control over your eating (at the time that you were eating)?
3. Over the past 28 days, on how many DAYS have such episodes of overeating occurred (i.e. you have eaten an unusually large amount of food and have had a sense of loss of control at the time)?
4. Over the past 28 days, how many times have you made yourself sick (vomit) as a means of controlling your shape or weight?
5. Over the past 28 days, how many times have you taken laxatives as a means of controlling your shape or weight?
6. Over the past 28 days, how many times have you exercised in a “driven” or “compulsive” way as a means of controlling your weight, shape or amount of fat or to burn off calories?

Questions 19-21: Please circle the appropriate number. Please note that for these questions the term “binge eating” means eating what others would regard as an unusually large amount of food for the circumstances, accompanied by a sense of having lost control overeating.

Response options:

- 1. No days
  2. 1-5 days
  3. 6-12 days
  4. 13-15 days
  5. 16-22 days
  6. 23-27 days
  7. Everyday

1. Over the past 28 days, on how many days have you eaten in secret (i.e., furtively)? Do not count episodes of binge eating.
2. On what proportion of the times you have eaten have you felt guilty (felt that you've done wrong) because of its effect on your shape or weight? Do not count episodes of binge eating.
3. Over the past 28 days, how concerned have you been about other people seeing you eat? Do not count episodes of binge eating.
4. Has your weight influenced how you think about (judge) yourself as a person?
5. Has your shape influenced how you think about (judge) yourself as a person?
6. How much would it have upset you if you had been asked to weigh yourself once a week (no more, or less, often) for the next four weeks?
7. How dissatisfied have you been with your weight?
8. How dissatisfied have you been with your shape?
9. How uncomfortable have you felt seeing your body (for example, seeing your shape in the mirror, in a shop window reflection, while undressing or taking a bath or shower)?
10. How uncomfortable have you felt about others seeing your shape or figure (for example, in communal changing rooms, when swimming, or wearing tight clothes)?
